# Outcomes of Bone Marrow-Derived Mononuclear Cell Transplantation for Patients in Persistent Vegetative State After Drowning: Report of Five Cases

**DOI:** 10.3389/fped.2020.00564

**Published:** 2020-09-10

**Authors:** Nguyen Thanh Liem, Vu Duy Chinh, Dam Thi Minh Phuong, Ngo Van Doan, Nicholas R. Forsyth, Michael Heke, Phuong Anh Nguyen Thi, Xuan-Hung Nguyen

**Affiliations:** ^1^Vinmec Research Institute of Stem Cell and Gene Technology (VRISG), Vinmec Health Care System, Hanoi, Vietnam; ^2^College of Health Sciences, VinUniversity, Hanoi, Vietnam; ^3^Vinmec Times City International Hospital, Vinmec Healthcare System, Hanoi, Vietnam; ^4^Institute for Science & Technology in Medicine, Keele University, Keele, United Kingdom; ^5^Department of Biology, Stanford University, Stanford, CA, United States

**Keywords:** drowning, persistent vegetative state, bone marrow mononuclear cells, autologous, cell therapy

## Abstract

**Aim:** Anoxic brain injury (ABI) due to non-fatal drowning may cause persistent vegetative state (VS) that is currently incurable. The aim of this paper is to present the safety and feasibility of autologous bone marrow-derived mononuclear cell (BMMNC) transplantation in five drowning children surviving in persistent VS.

**Methods:** We used BMMNC as a novel candidate therapeutic tool in a pilot phase-I study for five patients affected by neurological sequelae after near-death drowning. Autologous BMMNCs were freshly isolated using Ficoll gradient centrifugation then infused intrathecally to five patients. The number of transplantation varied from two to four times depending on the motor function improvement of patient after transplantation. Clinical therapeutic effects were evaluated using gross motor function measure and muscle spasticity rating scales, cognitive assessments, and brain MRI before and after cell administrations.

**Results:** Six months after BMMNC transplantation, no serious complications or adverse events were reported. All five patients displayed improvement across the major parameters of gross motor function, cognition, and muscle spasticity. Three patients displayed improved communication including the expression of words. In particular, one patient remarkably reduced cerebral atrophy, with nearly normal cerebral parenchyma after BMMNC transplantation.

**Conclusions:** Autologous BMMNC transplantation for the treatment of children in persistent VS after drowning is safe, feasible, and can potentially improve motor function and cognition and reduce muscle spasticity. These results pave the way for a future phase II clinical trial to evaluate the efficacy of the therapy.

## Introduction

Drowning is a major cause of mortality and neurological morbidity worldwide, accounting for 370,000 annual deaths in 2012 (1). Children aged 0–14 years are especially susceptible with over 450 children dying and thousands more suffering debilitating injuries every day due to drowning globally (2). For those children who survived drowning, anoxic brain injury (ABI)-induced neurological morbidity is a common outcome due to oxygen deprivation (3, 4). At least 5% of children admitted for drowning suffered from a severe neurological deficit, with outcomes of functional disability to vegetative states (VS) (2, 5).

From a pathological point of view, non-fatal drowning ABI involves a complex accumulation of brain injuries due to ischemia, hypoxia, cytotoxicity, and combinations thereof (6). These insults deprive the brain not only of oxygen but also of glucose and other nutrients required for neural metabolism. In severe non-fatal drowning incidents, the deprivation of these neural supporting supplements could trigger severe motor nerve deficits that largely underlie serious physical, cognitive, and behavioral complications (7). In these patients, quadriplegia resembling severe cerebral palsy is the most common consequence. Such morbidities cause a severe lifelong impact not only on the patients' health and well-being but also on their family (1), whereas no effective treatment is currently available.

There has been growing evidence of safety and benefit of autologous bone marrow-derived mononuclear cells (BMMNCs) in patients with a variety of neurological damage conditions (8–14). BMMNCs are a heterogeneous group of cells consisting of a small proportion of progenitor cells, such as hematopoietic stem cells (HSCs), mesenchymal stem cells (MSCs), MUSE cells, and endothelial progenitor cells that could potentially produce synergic effect promoting neuronal protection and regeneration and functional recovery.

Several characteristics make BMMNCs an attractive candidate for the management of VS. In animal models, there is evidence that transplanted BMMNCs cross the blood–brain barrier (15) and exert long-term neuroprotection by accelerating neuroplasticity and facilitating neuronal regeneration and functional recovery (16–19). In addition, they can promote endogenous neural stem cell (NSC) proliferation (20). Furthermore, high levels of growth factors, cytokines, and extracellular matrix molecules produced by BMMNCs could have potential neurotrophic or neuroprotective effects in the injured brain (21). Mesenchymal stem cells (MSCs), although representing a small fraction in BMMNCs, have been shown to differentiate into neuron-like cells and glial cells (22–25), whereby accelerating neuroplasticity, promoting neuronal regeneration and functional recovery, and enhancing the proliferation of endogenous neuronal stem cells (16–20). In addition, the presence of both MSCs and hematopoietic stem cells (HSCs) in BMMNCs could potentially produce a better and synergic effect as compared with each individual cell type (12–14). Besides, BMMNCs also contain MUSE cells that may contribute to neural cell regeneration and functional recovery (26–28).

In humans, we and others recently demonstrated the safety and feasibility of BMMNC transplantation in patients with a variety of neurological damage conditions such as traumatic brain injury (29, 30), cerebral hemorrhage (9), stroke (31–34), spinal cord injury (10, 11), cerebral palsy (12), and autism (13, 14). These encouraging results in combination with the neuroplasticity of the childhood brain provide a great opportunity for the stem cell-based treatment of pediatric non-fatal drowning ABI. The aim of this report is to present the safety and effectiveness of autologous BMMNC transplantation in five drowning children surviving in persistent VS.

## Patients and Methods

### Patient's Enrollment

We conducted a prospective case series using data collected from Vinmec International Hospital in Hanoi, Vietnam. The study was approved by the Hospital Board of Vinmec International Hospital. Five consecutive children with neurological sequelea, due to a near-death drowning incident were enrolled in this study. Clinical data was collected from January 2016 through December 2018. Following presentation of the Study Information Sheet, the participants' guardian(s) were made fully aware of the aim of the study and any potential side-effects before being invited to consider participant enrolment by signing the informed consent according to Good Clinical Practice and the Helsinki Declaration.

The general data collected before the transplantation consisted of age, gender, health condition prior to the accident, clinical findings concerning cognition, motor function, and muscle tone. All patients underwent neuroimaging assessments pre- and posttransplantation, using brain magnetic resonance imaging (MRI) on a 3-Tesla MRI device (Skyra Siemens, Germany) with the following sequence: axial T2W, FLAIR, IR, diffusion, ADCmap, coronal T2W, and sagittal T1W.

### Autologous BMMNC Collection, Isolation, and Transplantation

Autologous bone marrow was aspirated through an anterior iliac crest puncture under general anesthesia in the operating theater. The volume collected was in correlation with the patients' body weight, 8 ml/kg for patients under 10 kg [80 ml + (body weight in kg−10) × 7 ml] for patients above 10 kg (21).

Mononuclear cells were isolated from aspirated bone marrow by gradient centrifugation using Ficoll-Paque (GE Healthcare, Sweden) in a clean room following ISO 14644 standard at Vinmec Research Institute of Stem Cell and Gene Technology. The cell suspension was washed with phosphate-buffered saline (PBS) solution and resuspended in autologous plasma to a total volume of 10 ml for injection. The sterility of the product was confirmed by microbiological evaluation *via* the BacT/Alert® 3D microbial detection system (Biomerieux, USA). Total blood components before and after Ficoll-Plaque separation were evaluated by Beckman Coulter LH780 hemacytometer. The hematopoietic stem cell content (CD34^+^ cells) was assessed according to the International Society of Hematotherapy and Graft Engineering guideline (ISHAGE guideline) using Stem-Kit™ Reagent, Beckman Coulter in Navios flow cytometer. Before injection, cell products were examined for endotoxin levels with the Endosafe-PTS kit (Charles River).

Collected BMMNCs were infused intrathecally between the 4th and 5th lumbar vertebrae over the course of 30 min using an electrical pump, through a 22-gauge spinal needle. Each patient received a minimum of two times and maximum of four times of transplantations 6 months apart depending on his motor function improvement after transplantation. The number of BMMNCs that we collected each time is described in Table 1, and all the collected cells were used for transplantation. Based on our previous studies showing that BMMNC transplantation led to markedly improved gross motor function in patients with neurological complications such as cerebral palsy (12, 35) and neurological sequelae due to intracranial hemorrhage (36), the ability of patient to sit up was selected as satisfactory criteria to evaluate if the patient needs additional transplantation. If the patient could not show the improvement as indicated by their ability to sit up after BMMNC transplantation, additional transplantation will be implemented but not more than four times.

**Table 1:** General characteristics of the study participants at initial evaluation.

**Case**	**Gender**	**Age at transplantation** **(months)**	**Disease duration** **(months)**	**Modified ashworth scale**	**Transplantation**	**BMMNC** **(x 10^**6**^)/kg**	**CD34** **(x 10^**6**^)/kg**
1	M	26	3	4	1st	29.2	3.7
					2nd	38.2	5.3
					3rd	22.6	2.8
2	M	36	13	4–5	1st	10.9	0.6
					2nd	7.2	0.4
					3rd	24.6	1.3
					4th	18.8	1.2
3	M	63	4	5	1st	13.4	0.6
					2nd	33.5	5.5
					3rd	17.3	1.4
4	M	37	9	3–4	1st	33.8	7.6
					2nd	39.4	3.6
					3rd	87	5.0
5	M	25	2.5	4–5	1st	19.3	2.4
					2nd	52.3	4.4

After transplantation, physiotherapy by taking motor function exercises using motor-facilitation techniques was given to all the patient 2 h/day for 12 days at the hospital and then continued at home.

### Safety Evaluation

Adverse events were graded during and after BMMNC administration according to the NIH Common Terminology Criteria for Adverse Events (CTCAE), version 4 (https://ctep.cancer.gov/).

#### Efficacy Assessment

To evaluate the efficacy of BMMNC therapy, the muscular spasticity [Modified Ashworth Scale (MMAS)] (26), gross motor function (GMFM-88) (27), Denver II (Denver Developmental Screening Test) for neurological assessment (28), and German Coma Remission Scale (37) were measured before and at 6 and 12 months after transplantation. All patients were evaluated at admission and followed up after transplantations by a qualified rehabilitation specialist.

### Case Presentation

#### Case 1

**Figure 1:**
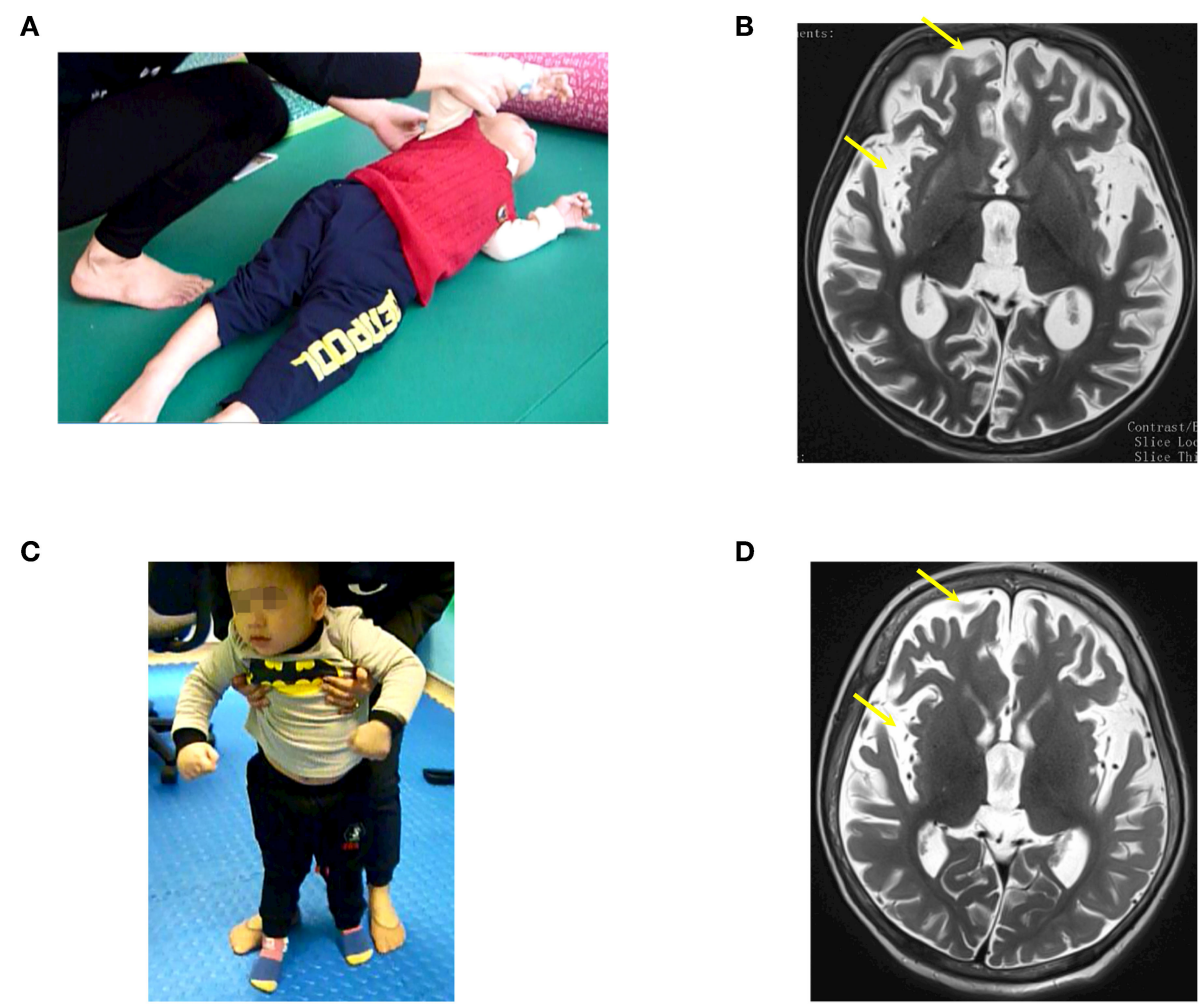
The patient (case 1) before and after autologous bone marrow-derived mononuclear cell (BMMNC) transplantation. **(A)** The patient before BMMNC transplantation is in a VS 3 months after the drowning. He had no awareness, no motor function, and exhibited muscle spasticity at level V. **(B)** Brain MRI demonstrated diffuse cerebral atrophy (arrows) at supratentorial and infratentorial regions, abnormal signal intensity at bilateral cortical parietal lobe, and bilateral putamen. **(C)** Six months after the third transplantation, the patient could express his need by vocalization, smile during communication, and stand. **(D)** No abnormal signal at bilateral cortical parietal lobe and bilateral putamen was observed at 12 months after the third transplantation.

A previous healthy 23-month-old male suffered from drowning with an estimated submersion period of 5 min. After drowning, he required a ventilator for 1 week. The duration from drowning to stem cell transplantation was 3 months. Before the accident, he could speak, run, and had good communication. After the accident, he had no awareness, no motor function, and exhibited muscle spasticity. Examination on 28th December 2016 when he was 26 months old showed a vegetative status with tetraplegia and generalized muscle spasticity of level V (Figure 1A). Total coma remission scale (CRS) was 5 points (Table 2) including arousability/attention of 1 point, motoric response of 1 point, response to acoustic stimuli of 0 point, response to visual stimuli of 1 point, response to tactile stimuli of 1 point, and logomotor (speech motoric) response of 1 point. Brain MRI demonstrated diffuse cerebral atrophy at the supratentorial and infratentorial regions and abnormal signal (increased signal intensity) at the bilateral cortical parietal lobe and bilateral putamen (Figure 1B).

**Table 2:** Summary of clinical parameters of our five-patient cohort.

**Case**	**Motor function after** **the last transplantation**	**Cognition after the** **last transplantation**	**Changes of** **muscle spasticity**	**Coma remission scale**	
				**Before transplantation**	**After the last transplantation**
1	Sit up	Speak	Levels V to I	5	22
2	Turn	Express emotions	Levels V to II	4	20
3	Sit up	Express emotions	Levels V to III	8	24
4	Sit up and crawl	Speak	Levels IV to I	6	22
5	Sit up	Speak	Levels V to II	3	15

The patient underwent three BMMNC transplantations without any adverse events. Cellular information is presented in Table 1.

##### Progress after transplantations

Improvements of cognition, motor functions, and muscle tonus were observed after each transplantation. Following on from the first transplantation, he could express emotion during communication, make some sounds, and turn. Total CRS increased to 15 points including 3 points in arousability/attention, 4 points in motoric response, 2 points in response to acoustic, 2 points in response to visual stimuli, 2 points in response to tactile stimuli, and 2 points in logomotor (speech motoric) response. The second transplantation saw improvements in response to sound and light, eye movement in response to objects, and sitting with support. Total CRS increased to 19 points including 4 points in arousability/attention, 5 points in motoric response, 3 points in response to acoustic stimuli, 3 points in response to visual stimuli, 2 points in response to tactile stimuli, and 2 points in logomotor (speech motoric) response.

The third transplantation resulted in an ability to express his need by vocalization, to smile during communication, and to stand without support (Figure 1C). Muscle spasticity was reduced from level V to level I after three transplantations. Total CRS increased to 22 points (Table 2).

A brain MRI taken on 11th January 2018 (12 months after the first transplantation) showed that diffuse cerebral atrophy at the supratentorial and infratentorial regions still existed. However, there was no abnormal signal at the bilateral cortical parietal lobe and bilateral putamen (Figure 1D).

#### Case 2

**Figure 2:**
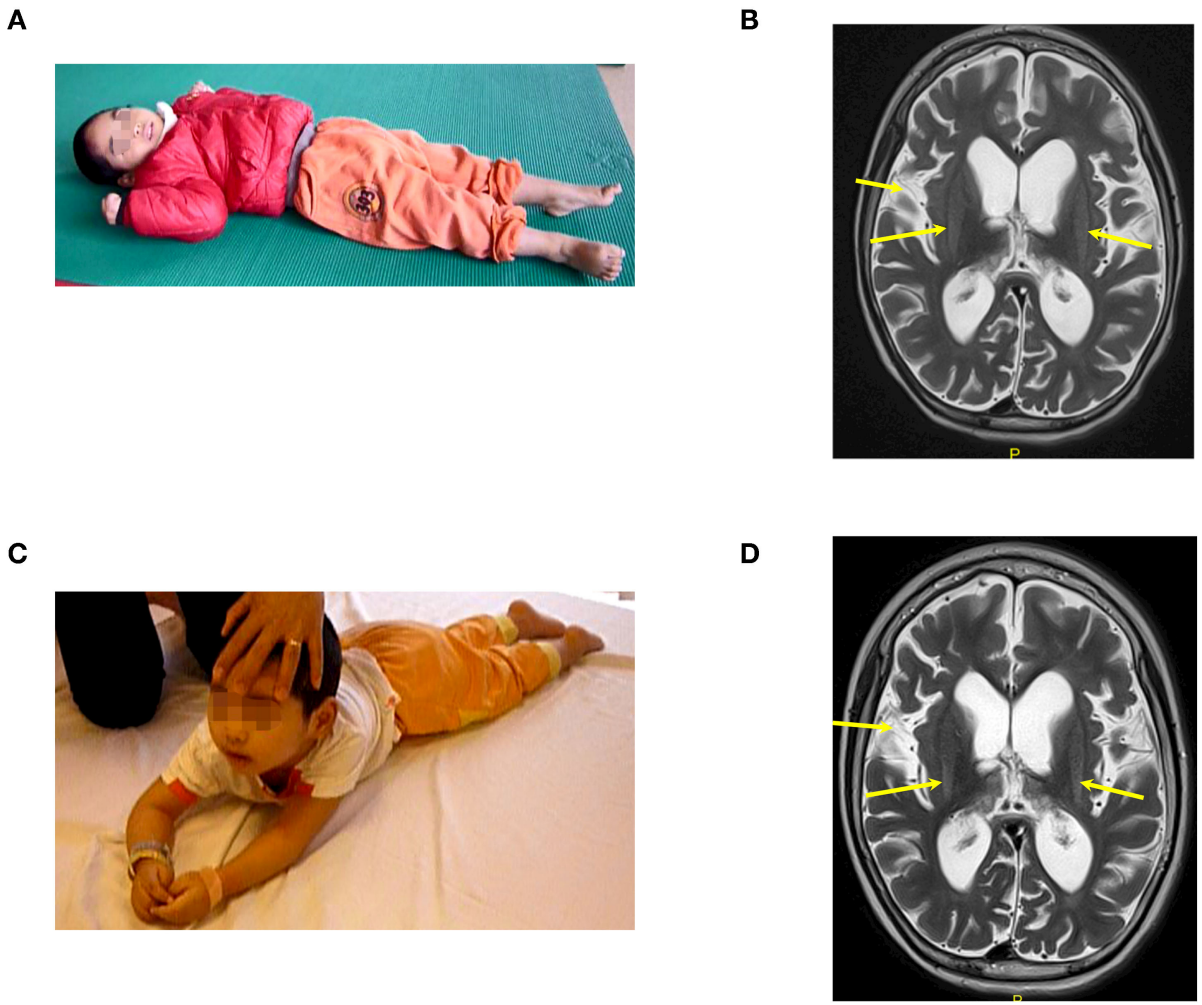
The patient (case 2) before and after autologous BMMNC transplantation. **(A)** Thirteen months after the drowning before BMMNC transplantation, the patient is in a VS without awareness, no motor function, and exhibited muscle spasticity at level V. **(B)** Diffuse cerebral atrophy at supratentorial region and abnormal signal at bilateral putamen on brain MRI before transplantation (arrows). **(C)** Six months after the fourth transplantation, the patient could respond to sound and music, smile or cry during communication, and turn and lift his head up when lying prone. **(D)** However, there was no significant change in brain MRI after transplantations in comparison with that before transplantation.

Case 2, a male, born on 27th January 2013, suffered from drowning at 23 months old. His estimated duration in the water was approximately 10 min. After drowning, he required a ventilator for 7 weeks. The duration from drowning to BMMNC transplantation was 13 months. Before drowning, he could speak, understand, walk, run, and had good communication. After drowning, he lost his awareness and motor function. Examination on 25th January 2016, when he was 36 months old, confirmed his vegetative status: no awareness, no motor function, and generalized muscle spasticity at level V (Figure 2A). Total CRS was only 4 points (Table 2) including 1 point in arousability/attention, 1 point in motoric response, 1 point in response to acoustic stimuli, 0 point in response to visual stimuli, 0 point in response to tactile stimuli, and 1 point in logomotor (speech motoric) response. Brain MRI showed diffuse cerebral atrophy at the supratentorial region and abnormal signal at the bilateral putamen (Figure 2B).

The patient underwent four BMMNC transplantations with cellular information as presented in Table 1. No adverse effects were observed. After discharge, a standard physiotherapy regimen was applied as described for patient 1.

##### Progress after transplantations

After transplantations, improvements of cognition were observed. Emotional expression when being called was noted after the first transplantation and cognition continued to improve progressively. Initial improvement of total CRS was noted with an increase of total score to 10 points including 2 points in arousability/attention, 3 points in motoric response, 1 point in response to acoustic stimuli, 1 point in response to visual stimuli, 2 points in response to tactile stimuli, and 1 point in logomotor (speech motoric) response.

Following on from the fourth transplantation, he could respond to sound and music and could smile or cry during communication. Improvement in motor function was also observed. After four transplantations, he could turn and could lift his head up when lying prone (Figure 2C). Muscle spasticity was reduced from level V to level II. Total CRS reached to 20 points (Table 2) including 4 points in arousability/attention, 5 points in motoric response, 3 points in response to acoustic stimuli, 3 points in response to visual stimuli, 3 points in response to tactile stimuli, and 2 points in logomotor (speech motoric) response.

Brain MRI taken on 16th December 2017 (23 months after the first transplantation) showed no significant change when compared with the MRI findings before the transplantation (Figure 2D).

#### Case 3

Case 3, a male, born on 26th March 2011, suffered from drowning at 59 months old. His estimated duration in the water was ~5 min. After cardiopulmonary resuscitation, he required a ventilator for 5 weeks. The duration from drowning to the first transplantation was 4 months.

**Figure 3:**
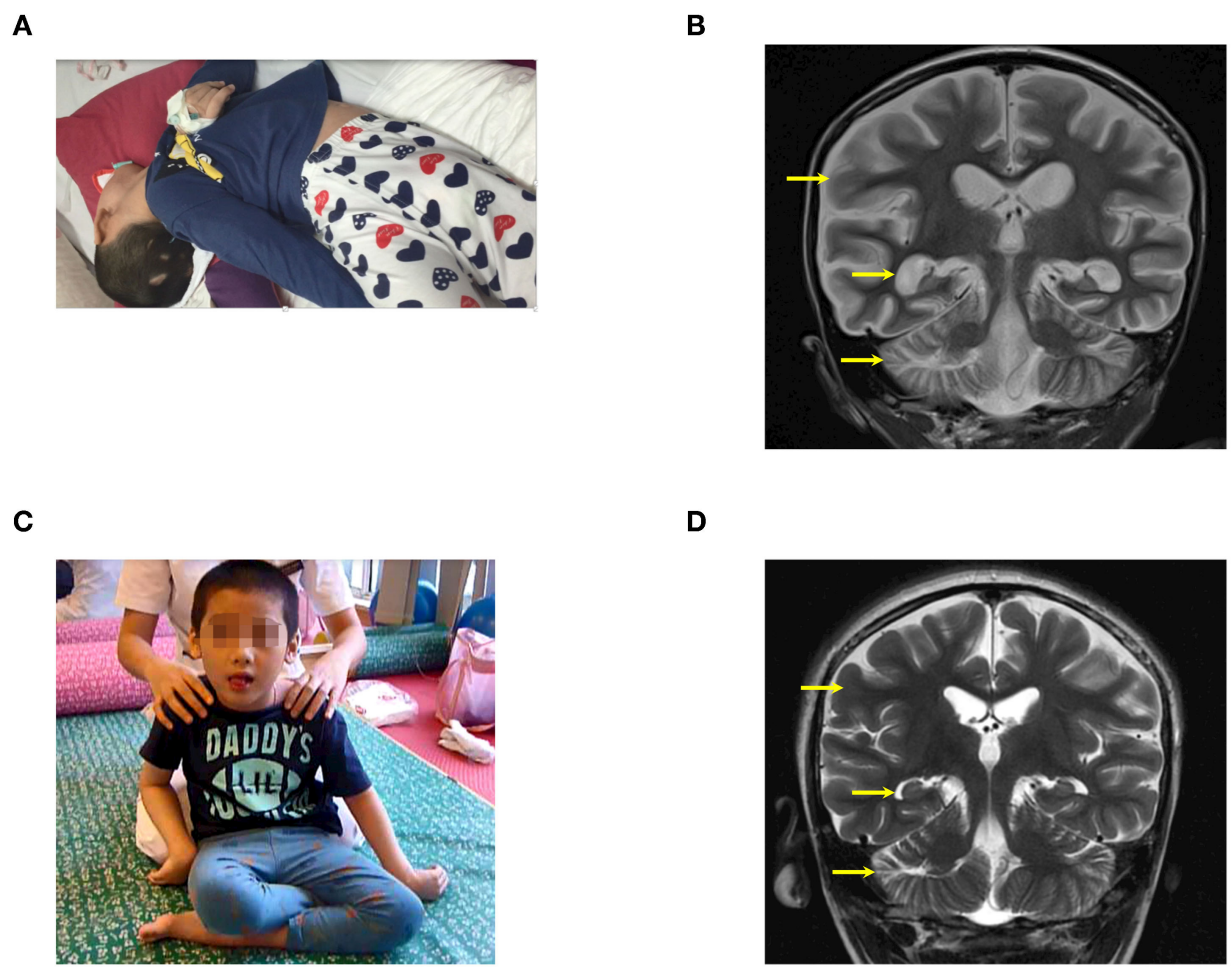
The patient (case 3) before and after autologous BMMNC transplantation. **(A)** The patient before BMMNC transplantation is in a VS 4 months after the drowning. He had no awareness and suffered from quadriplegia with muscle spasticity at level V. **(B)** Diffuse cerebral atrophy at the supratentorial and infratentorial regions on brain MRI before transplantation (arrows). **(C)** Six months after the third transplantation, the patient could respond to calling, recognized objects, sit up, and expressed feelings. **(D)** Brain MRI showed a remarkably reduced cerebral atrophy with near-normal cerebral parenchyma (arrows) after stem cell transplantation.

Prior to the accident, his cognition, communication and motor function had developed normally. Examination on 3rd May 2016, when he was 63 months, revealed that the child had no awareness and suffered from quadriplegia and generalized muscle spasticity at level V, causing an abnormal posture of the body, legs, feet, hands, and forearm (Figure 3A). Total CRS was 8 points (Table 2) with 2 points in arousability/attention, 2 points in motoric response, 1 point in response to acoustic stimuli, 1 point in response to visual stimuli, 1 point in response to tactile stimuli, and 1 point in logomotor (speech motoric) response. Epilepsy occurred more than 10 times/day, and its treatment required 500 mg of Keppra and 200 mg of Depakin daily.

Brain MRI showed diffused cerebral atrophy at the supratentorial and infratentorial regions (Figure 3B). His electroencephalogram (EEG) showed diffused epileptic waives. The child received three transplantations at 63, 69, and 76 months of age, without any adverse effects. Cell details are presented in Table 1.

##### Progress after transplantation

After three transplantations, cognition was significantly improved, the child responded to calling, recognized objects, expressed need, expressed feelings (such as happiness or sadness), and watched TV. During the last examination, he could reach a maximal CRS of 24/24 points (Table 2) including 5 points in arousability/attention, 6 points in motoric response, 3 points in response to acoustic stimuli, 4 points in response to visual stimuli, 3 points in response to tactile stimuli, and 3 points in logomotor (speech motoric) response. Muscle spasticity reduced from level V to level III. The child could also sit up (Figure 3C) with no manifestation of epilepsy; however, medication with Keppra was still maintained at 500 mg/day.

Brain MRI result shows that cerebral atrophy was reduced remarkably compared with the brain MRI before transplantation, with nearly normal cerebral parenchyma (Figure 3D).

#### Case 4

Case 4, a male, born on 26th July 2013, suffered from drowning at 28 months old. His estimated time in the water was ~10 min. He required a ventilator for 10 days. The duration from drowning to stem cell transplantation was 9 months.

**Figure 4:**
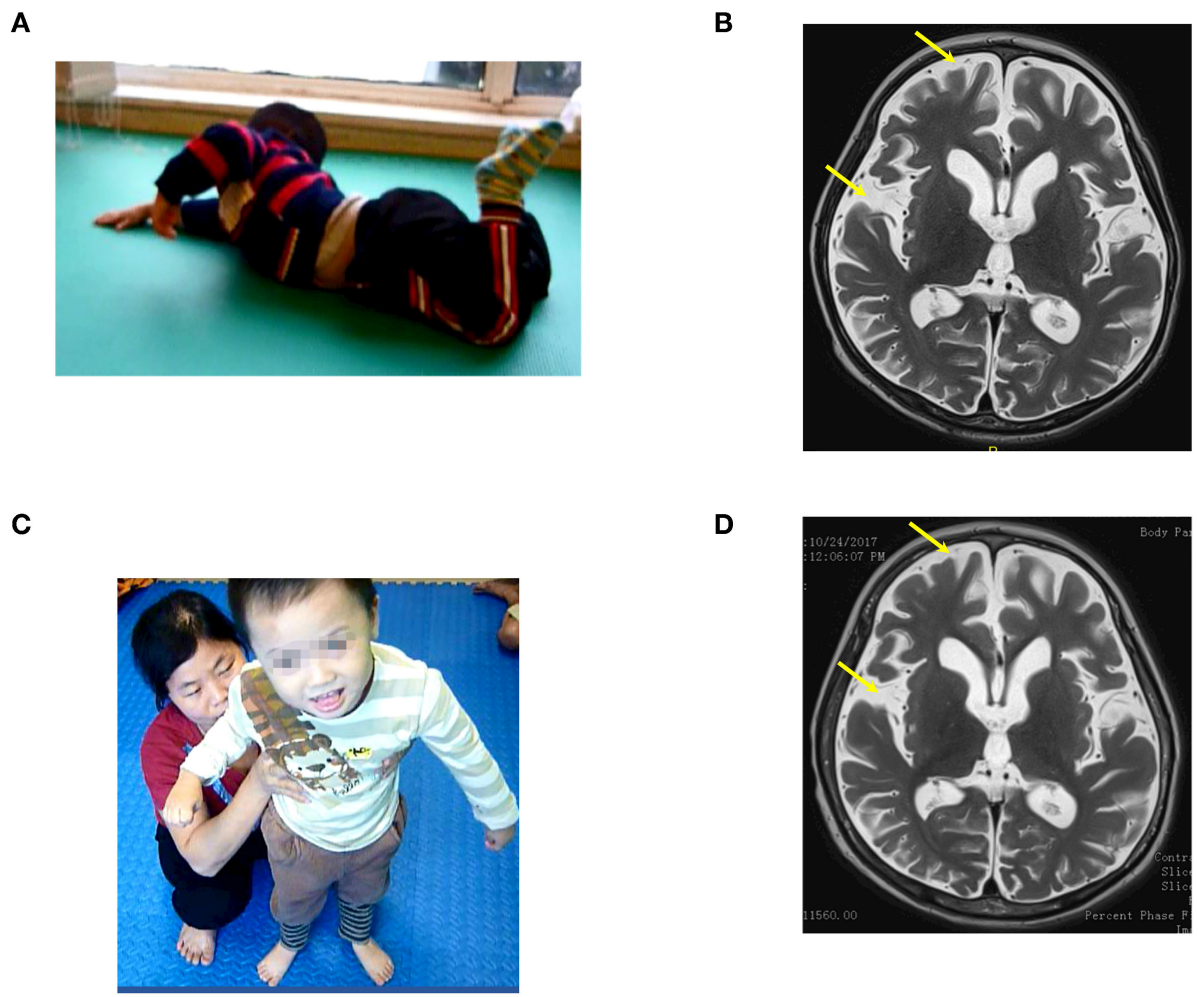
The patient (case 4) before and after autologous BMMNC transplantation. **(A)** Nine months after the drowning before BMMNC transplantation, the patient had no reaction during communication and could not follow objects and speak and exhibited muscle spasticity at level V. He could not sit up or crawl and had epilepsy. **(B)** Brain MRI revealed diffuse cerebral atrophy before transplantation (arrows). **(C)** Six months after the third transplantation, the patient's cognition improved progressively and could stand with support. **(D)** However, brain MRI showed no significant change after transplantations.

Before the accident, the child attended nursery school. He could speak and sing simple songs, and his motor function and communication had developed normally. At examination on 16th August 2016, before the transplantation when he was 37 months old, his social interaction was equivalent to a child at 3 months of age. He could not recognize his parents, had no reaction during communication, could not follow objects, and he could not speak. Total CRS was 6 points (Table 2) as follows: 1 point in arousability/attention, 1 point in motoric response, 1 point in response to acoustic stimuli, 1 point in response to visual stimuli, 1 point in response to tactile stimuli, and 1 point in logomotor (speech motoric) response. He could turn while lying on the floor but could not sit up or crawl (Figure 4A). Muscle spasticity was assessed at level IV. Epilepsy occurred and he required 100 mg of Depakin, 1–2 times/day.

Brain MRI on 6th July revealed a diffused cerebral atrophy (Figure 4B). EEG revealed diffused epileptic waves.

He received three BMMNC transplantations at the age of 37, 43, and 49 months (Table 1), respectively, without any adverse events.

##### Progress after transplantations

Improvements in cognition, motor function, and muscle spasticity were manifested after transplantations. Cognition improved progressively. He could recognize his parents, smile during communication, and make some sounds, subsequent to the last transplantation. The last examination revealed that total CRS was 22 points (Table 2) including 4 points in arousability/attention, 5 points in motoric response, 3 points in response to acoustic stimuli, 4 points in response to visual stimuli, 3 points in response to tactile stimuli, and 3 points in logomotor (speech motoric) response. His motor function also improved. After the first transplantation, the patient could sit up with support. He could sit without support and could crawl after the second transplantation, and eventually could stand with support following the last transplantation (Figure 4C). Muscle spasticity reduced from level IV to level I after the transplantations. Epilepsy no longer occurred after the first transplantation. Medication with Depakin was gradually reduced and eventually suspended without recurrence of epilepsy.

Brain MRI taken on 24th October 2017 showed no significant changes compared with the findings before the first transplantation (Figure 4D).

#### Case 5

Case 5, a male, born on 18th August2015, suffered from drowning at the age of 23 months. His estimated duration in the water was approximately 5 min. After drowning, he required a ventilator for 7 days. The duration from drowning to stem cell transplantation was 2.5 months.

**Figure 5:**
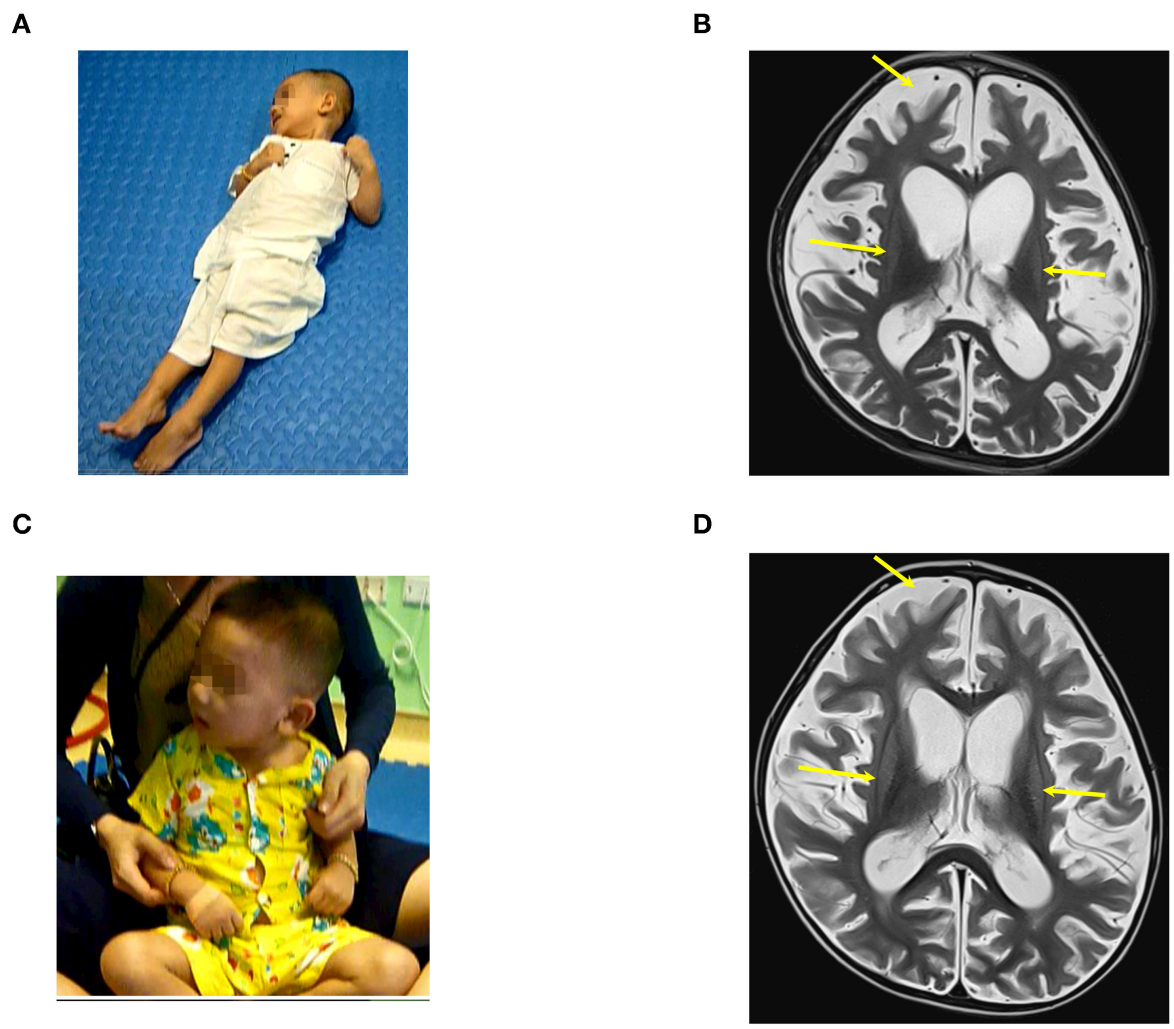
The patient (case 5) before and after autologous BMMNC transplantation; **(A)** 2.5 months after the drowning before BMMNC transplantation, the patient is in a VS. without awareness, no motor function, having tetraplegia, and exhibited muscle spasticity at level V. **(B)** Diffuse brain atrophy and abnormal signal at bilateral putamen on brain MRI before transplantation (arrows). **(C)** Six months after the second transplantation, the patient could display limited vocal capacity and turn and sit up with support. **(D)** There was no significant change in brain MRI after transplantations compared with before transplantation.

Prior to drowning, he had started to speak and had normal motor function and cognition. Examination on 25th September 2017, when he was 25.5 months old, confirmed a vegetative status. There was no awareness or motor function, and the child had tetraplegia and generalized muscle spasticity at level V (Figure 5A). Total CRS was only 3 points (Table 2) with arousability/attention of 0 point, motoric response of 1 point, response to acoustic stimuli of 0 point, response to tactile stimuli of 1 point, and logomotor (speech motoric) response of 1 point.

Brain MRI on 23rd August 2017 showed a diffuse brain atrophy and abnormal signal at the bilateral putamen (Figure 5B).

He underwent two BMMNC transplantations at 25 and 32 months without any adverse events. Details about the transplanted cells are presented in Table 1.

##### Progress after transplantation

The first transplantation resulted in a capacity to respond to sounds and voices. Total CRS increased to 10 points including 2 points in arousability/attention, 3 points in motoric response, 2 points in response to acoustic stimuli, 1 point in response to visual stimuli, 1 point in response to tactile stimuli, and 1 point in logomotor (speech motoric) response. Following on from the second transplantation, he could recognize his parents, interact with media, display limited vocal capacity, and turn and sit up with support (Figure 5C). The last examination showed that total CRS reached 15 points (Table 2) including 3 points in arousability/attention, 4 points in motoric response, 2 points in response to acoustic stimuli, 2 points in response to visual stimuli, 2 points in response to tactile stimuli, and 2 points in logomotor (speech motoric) response.

Muscle spasticity reduced from level V to level II after two transplantations. However, no significant changes on brain MRI were observed 9 months after the first transplantation (Figure 5D).

A summary of clinical parameters for the five patients are presented in Table 2.

## Discussion

To our knowledge, this is the first publication using BMMNC transplantation in the treatment of neurologic sequelea after near-death drowning. BMMNC transplantation-related adverse events were not observed, indicating the safety of BMMNC transplantation in patients with neurologic sequelae after drowning.

In a neurologic long-term follow-up study of children that suffered severe near-death drowning, the vegetative survivors displayed no significant improvement after hospital discharge (38). At present, there is no treatment for children in persistent VS after drowning. Here, we found that BMMNC transplantation clearly improved the major parameters of gross motor function, cognition, and muscle spasticity in the five cases of VS after drowning. Improvements in motor function occurred in all patients. Two patients could sit up (case 1 and 4) and two others (case 3 and 5) could stand up after transplantation. Muscle spasticity was significantly reduced after transplantations which contributed to improvements of motor function. Remarkable improvements of cognition were observed in all five patients. Especially, three patients (cases 1, 4, and 5) not only had better communication but could also express words. Until January 2020, all the BMMNC transplant recipients are in stable conditions when followed up by telephone consultation.

Remarkably, in two patients with epilepsy, associated epilepsy abated in one patient (case 4) and ceased completely in another one (case 3) after BMMNC transplantation, thereby indicating that BMMNC transplantation may ameliorate epileptic status in patients with neurologic sequelae after drowning. To our knowledge, this is the first report demonstrating a potential role of BMMNC transplantation in ameliorating epileptic status in patients with neurological complications after near-death drowning.

Clinical improvements were observed in all five cases after BMMNC transplantation. However, changes in brain tissue were only observed in two patients after BMMNC transplantation (cases 1 and 3), suggesting that functional recovery could be more obviously observed than morphologic improvement of the brain.

Autologous BMMNCs are a blend of undifferentiated cells, including stem and progenitor cells that can be readily isolated from bone marrow without ethical and technical concerns. For the treatment of neurological deficits, it may be advised to use BMMNCs as a heterogeneous cell mixture, in which some cells may repair nerves through cell replacement, while other cells may secrete different cytokines, growth factors, and extracellular matrix molecules to assist in nerve repair and regeneration, thereby supporting the growth of NSCs and inducing NSC differentiation (39). In summary, cell therapy with autologous BMMNC appeared to be safe and feasible for VS patients. We observed potential effect of BMMNC transplantation on functional recovery in the patients with neurologically devastated by drowning, although the study was not designed with control to draw that conclusion.

There are several limitations in this study. First, this was a case series with small number of patients and designed without controls. Indeed, there could be an isolated or added volumizing effect in these patients. The small number of patients in this study also makes it difficult to determine the risk factors related to outcomes. Second, the patients had been recruited into our hospital for BMMNC transplantation after receiving the emergency care at the local hospitals, leading to the insufficient clinical records related to drowning-related complications. Third, two to four times of BMMNC transplantations have been done based on the ability of the patients to sit up after transplantation, and the first transplantation was performed at different time points after the disease onset across patients, whether a different in number of transplantation and time after disease onset would have been associated with different outcomes cannot be determined. Fourth, quantitative assessments to evaluate the vegetative state, the gross motor function and recovery of speech were not performed in this study. Together, a prospective study with a greater cohort of participants and quantitative functional assessment in a randomized controlled trial should be performed to draw accurate and solid conclusions about efficacy of BMMNC transplantation to improve patient's functions after near-death drowning.

## Conclusions

Our results showed that BMMNC transplantation was safe and could improve motor function and cognition of patients with neurological sequelea after near-death drowning.

## Data Availability Statement

All datasets generated for this study are included in the article/supplementary material.

## Ethics Statement

The studies involving human participants were reviewed and approved by Hospital Board of Vinmec International Hospital in Hanoi. Written informed consent to participate in this study was provided by the participants' legal guardian/next of kin. Written informed consent was obtained from the individual(s), and minor(s)' legal guardian/next of kin, for the publication of any potentially identifiable images or data included in this article.

## Author Contributions

NL: study concept and design. All authors: data collection and analysis. VC and NV: provision of study materials or patients. NL, NF, MH, and X-HN: drafting the manuscript. All authors critically reviewed and approved the final manuscript.

## Conflict of Interest

The authors declare that the research was conducted in the absence of any commercial or financial relationships that could be construed as a potential conflict of interest.

## Abbreviations

ABI: Anoxic brain injury
BMMSC: Bone marrow-derived mesenchymal stem cell
BMMNC: Bone marrow-derived mononuclear cell
CRS: Coma remission scale
EGG: Electroencephalogram
HSC: Hematopoietic stem cell
MRI: Magnetic resonance imaging
MSC: Mesenchymal stem cell
PBS: Phosphate-buffered saline
VS: Vegetative state
NSC: Neural stem cell.
